# Traumatic brain injury alters the relationship between brain structure and episodic memory

**DOI:** 10.1002/brb3.3012

**Published:** 2023-05-03

**Authors:** Abbie S. Taing, Matthew E. Mundy, Jennie L. Ponsford, Gershon Spitz

**Affiliations:** ^1^ School of Psychological Sciences, Turner Institute for Brain and Mental Health Monash University Clayton Victoria Australia; ^2^ Monash Epworth Rehabilitation Research Centre Richmond Victoria Australia; ^3^ Faculty of Health and Education Torrens University Melbourne Victoria Australia

**Keywords:** cortical thickness, emergent memory account, episodic memory, MRI, traumatic brain injury

## Abstract

**Background:**

Focal and diffuse pathology resulting from traumatic brain injury (TBI) often disrupts brain circuitry that is critical for episodic memory, including medial temporal lobe and prefrontal regions. Prior studies have focused on unitary accounts of temporal lobe function, associating verbally learned material and brain morphology. Medial temporal lobe structures, however, are domain‐sensitive, preferentially supporting different visual stimuli. There has been little consideration of whether TBI preferentially disrupts the type of visually learned material and its association with cortical morphology following injury. Here, we investigated whether (1) episodic memory deficits differ according to the stimulus type, and (2) the pattern in memory performance can be linked to changes in cortical thickness.

**Methods:**

Forty‐three individuals with moderate‐severe TBI and 38 demographically similar healthy controls completed a recognition task in which memory was assessed for three categories of stimuli: faces, scenes, and animals. The association between episodic memory accuracy on this task and cortical thickness was subsequently examined within and between groups.

**Results:**

Our behavioral results support the notion of category‐specific impairments: the TBI group had significantly impaired accuracy for memory for faces and scenes, but not animals. Moreover, the association between cortical thickness and behavioral performance was only significant for faces between groups.

**Conclusion:**

Taken together, these behavioral and structural findings provide support for an emergent memory account, and highlight that cortical thickness differentially affects episodic memory for specific categories of stimuli.

## Graphical abstract

The current study investigated whether TBI preferentially disrupts the type of visually learned material and its association with cortical morphology following injury. Behavioural results support the notion of category‐specific impairments. Cortical thickness was also differentially affected depending on the specific categories of stimuli.

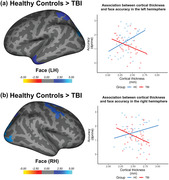

## 1 INTRODUCTION

Traumatic brain injury (TBI) causes both focal and diffuse brain pathology (Bigler, 2001), resulting in a diverse range of functional deficits (Strangman et al., 2010). Given preferential disruption to frontotemporal brain regions, impairments in episodic memory—defined as the ability to learn, store, and retrieve information about personal experiences (Moscovitch, 1995)—are among the most frequently reported symptom (Vakil, 2005).

It is well established that episodic memory relies on a distributed brain network, comprising regions within the frontal, temporal, and parietal lobes (Dickerson & Eichenbaum, 2010; Eichenbaum, 2017; Moscovitch et al., 2016; Wagner et al., 2005). Medial temporal structures are critical for the formation and retrieval of episodic memory (Dickerson & Eichenbaum, 2010; Simons & Spiers, 2003). Prefrontal cortical regions, the temporoparietal junction, and retrosplenial/posterior cingulate cortex also support various memory processes, including working memory, attention, and basic mnemonic processes (Svoboda et al., 2006).

The relationship between brain structure and episodic memory following TBI has been examined in several studies. Overall, studies have reported a relationship between disrupted cortical morphology and impaired episodic memory (Ariza et al., 2006; Bigler et al., 2002; Govindarajan et al., 2016; Levine et al., 2013; Palacios et al., 2013; Santhanam et al., 2019; Spitz et al., 2013; Strangman et al., 2010; Wang et al., 2015). These studies, however, have largely examined memory within a traditional, unitary, account of temporal lobe function. Instead, medial temporal lobe structures are domain‐sensitive, preferentially supporting encoding and retrieval processes for different visual material (Graham et al., 2010). For example, processing of faces robustly activates regions in the middle fusiform gyrus (“fusiform face area”), lateral inferior occipital gyrus (“occipital face area”), and superior temporal sulcus (Haxby et al., 2002; Hoffman & Haxby, 2000; Kesler et al., 2000); processing of scenes activates the posterior parahippocampus (Epstein & Ward, 2010); and processing of animals activates the bilateral fusiform gyrus (Downing et al., 2006; Rogers et al., 2005). Accordingly, stimulus‐specific memory impairments may arise following damage to the medial temporal lobe. Indeed, category‐specific impairments for complex stimuli such as faces and scenes have been reported in amnesia patients with selective medial temporal lobe damage (Mundy et al., 2013; Taylor et al., 2007).

Here, we use a combined behavioral and structural MRI paradigm to examine whether the relationship between episodic memory and cortical thickness differs depending on stimulus type. To first establish the presence of category‐specific impairments, an episodic memory task (Taing et al., 2021) was used to assess whether recognition of prior learned visual stimuli was differentially affected for three categories of stimuli: faces, scenes, and animals. The association between performance on this behavioral task and cortical thickness was subsequently examined within and between groups. We hypothesized that the TBI group would show impaired recognition memory for complex stimuli such as faces and scenes. In line with previous studies, we further hypothesized that the TBI group would have lower cortical thickness compared to healthy controls. Finally, we hypothesized that the accuracy of prior visually learned material would be associated with lower cortical thickness in regions known to support processing of the specific stimuli. More specifically, we hypothesized that cortical thickness in medial temporal and occipital structures would differentially affect episodic memory for faces, scenes, and animals.

## 2 MATERIAL AND METHODS

### 2.1 Participants

The study was approved by Monash Health/University Human Research Ethics Committee and conducted in accordance with the Declaration of Helsinki. Written informed consent was obtained from all participants. Forty‐three individuals (31 males, 12 females) with moderate to severe TBI, as defined by post‐traumatic amnesia (PTA) duration > 1 day (*M* = 26.9 day, *SD* = 28.06 days) measured using the Westmead Post Traumatic Amnesia Scale (WPTAS), participated in the study. The average age and years of education were 40.8 years (*SD* = 16.46) and 14.2 years (*SD* = 2.94), respectively (Supplementary Table 1 and 2). To determine whether memory impairment was affected across varying recovery time and pathology, TBI participants were recruited at an average of 11 months post injury (*SD* = 11.57 months, range = 0.69–34.82 months) and had mixed focal and/or diffuse pathology (Supplementary Figure 1).

Thirty‐eight healthy controls of similar age (*M =* 40.8 years, *SD* = 16.46 years), sex (26 male/12 female), and education (*M* = 14.7 years, *SD* = 2.79 years) were also recruited. No significant group differences were apparent on any of the demographic variables (*p* > .05). Exclusion criteria included age < 18 or > 75 years, prior history of TBI or other neurological conditions, significant psychiatric or substance abuse history, and MRI contraindication. Three TBI participants were excluded from the behavioural analysis due to a technical error during task administration.

### 2.2 Episodic memory paradigm

An episodic memory paradigm (Taing et al., 2021) was used to assess episodic encoding and recognition. During encoding, participants were presented with three categories of stimuli (i.e., faces, scenes, and animals) and asked to respond to these stimuli based on set criteria (e.g., decide whether an animal is shorter or taller than a human man; whether a face is male or female; whether a scene looks hot or cold) to ensure attention was maintained throughout the task. This task consisted of six blocks, with each block containing 20 images from each of three stimulus categories. To ensure equivalent task difficulty, stimulus presentation was reduced for individuals recruited further along post injury (each stimulus was presented for 3 s and followed by a 3 s interstimulus interval for those less than 1 year post injury; or stimulus presentation of 2 s and followed by a 2 s interstimulus interval for those more than 1 year post injury). Five rest blocks were presented after each experimental block.

To assess the accuracy of prior learned material, participants were required to classify images as “old” (i.e., images seen during the encoding phase) or “new” (i.e., images not seen during the encoding phase) approximately 30 min following stimuli encoding. Participants rated a total of 60 old and 60 new stimuli over two presentation runs (240 stimuli in total). Furthermore, participants rated all old and new stimuli during the first presentation run before having to rate each stimulus again. Performance on the first presentation run is thus a purer marker of episodic memory (since the second presentation run also assesses source memory) and therefore was the primary outcome measure used in the analysis. Additional information for the overall performance (i.e., first and second presentation runs combined) can be found in the Supplementary Material (see “Episodic Memory (Overall) and Cortical Thickness” and Supplementary Figure S2).

### 2.3 MRI acquisition

Structural MR images were acquired across three different sites, all comprising 3.0 Tesla Siemens Magnetom Skyra scanners. The following T1‐weighted image parameters were used in two of the sites: repetition time (TR) = 2.3 s; echo time (TE) = 2.32 ms; flip angle = 8°; 236 × 350 matrix; voxel size = 0.9 × 0.9 × 0.9 m. The third site had the following parameters: TR = 2.0 s; TE = 2.03 ms; flip angle = 8°; 256 × 256 matrix; voxel size = 1.0 × 1.0 × 1.0 mm.

### 2.4 Statistical analysis

#### 2.4.1 Behavioral and demographic data

Behavioral and demographic data were analysed using R version 3.6.0 (R Core Team). Independent samples t‐tests were used to assess group differences on the demographic variables (i.e., age, sex, and years of education). Behavioral data were screened and assessed for violation of statistical assumptions prior to analysis. The behavioral measure of interest was accuracy as determined using dprime—a sensitive index which measures an individual's ability discriminate signal from noise. Linear mixed models were used to analyse accuracy to better account for clustering and nonindependence of measures within participants. Task accuracy was assessed by modeling group, stimulus category, and their interaction (group × stimulus category) as fixed effects, and participant as a random effect. Age and education were added as covariates in the model given they could affect episodic memory performance (Hoyer & Verhaeghen, 2006; Lachman et al., 2010). Given that we expected lower performance for the TBI group, post hoc analyses were followed up using one‐tailed independent samples t‐tests with multiple comparison correction.

#### 2.4.2 Imaging data

Cortical reconstruction and segmentation were performed with Freesurfer (http://surfer.nmr.mgh.harvard.edu) using the processing pipeline implemented within fMRIPrep 20.0.039 (Esteban et al., 2019). This involved several stages including spatial normalization, brain tissue segmentation, and surface reconstruction—the technical details of which are described elsewhere (see Fischl, 2012). Prior to conducting statistical analyses, quality control of the Freesurfer registration and segmentation was undertaken by visual inspection to ensure that proper classification has occurred. Misclassified areas were manually corrected and the Freesurfer steps were rerun. Further information about the pipeline can be found in the Supplementary Material (see “Detailed MRI Preprocessing”).

The General linear model (GLM) for surface‐based analysis was used to model cortical thickness using Freesurfer. Analyses were undertaken in several stages. We first determined whether overall differences in cortical thickness between the TBI group and healthy controls. Subsequently, the association between cortical thickness and memory performance was examined both within and between groups (i.e., one‐sample and two‐sample *t*‐tests with memory performance as a covariate). The following parameters, adjusted for multiple comparisons, were used: smoothing = 10, the inclusion of age, years of education, and site as nuisance covariates, Monte–Carlo simulation threshold of 1.3 (corresponding to a false‐discovery rate of *p* < .05), and the appropriate one‐tailed test (i.e., positive or negative) was applied.

## 3 RESULTS

### 3.1 Episodic memory is impaired for complex stimuli such as faces and scenes following TBI

**FIGURE 1:**
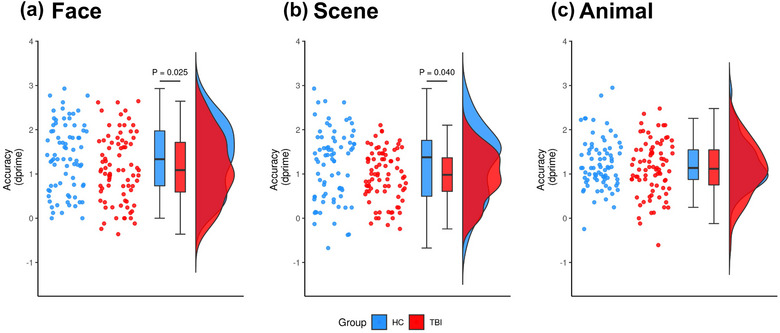
The TBI group was significantly less accurate than healthy controls on the episodic memory task, as measured using dprime (higher values denote better performance). Plots show individual datapoints along with boxplots and violin plots showing distribution. Post hoc analyses indicated that the TBI group (red) had significantly poorer accuracy than healthy controls (blue) for faces (*p* = .025; A) and scenes (*p* = .040; B). There was no significant difference in accuracy between the groups for animals (*p* > .05; C). *Note*: Reported *p* values were adjusted for multiple comparisons.

Overall, the TBI group was significantly less accurate than healthy controls across all stimuli types (*t*(224) = −2.02, 95% CI [−0.60 to −0.009], *p* = .022). Post hoc analyses indicated worse accuracy for the TBI group compared to healthy controls for faces (*t*(177) = −1.98, 95% CI [−0.61 to −0.001]; *p* = .025; Figure 1A) and scenes (*t*(177) = −1.77, 95% CI [−0.57 to 0.03]; *p* = .040; Figure 1B). There was also a trend for lower accuracy for animals, although this did not reach statistical significance (*t*(177) = −1.26, 95% CI [−0.50 to 0.11]; *p* = .105; Figure 1C).

### 3.2 Lower cortical thickness evident for individuals with TBI

**FIGURE 2:**
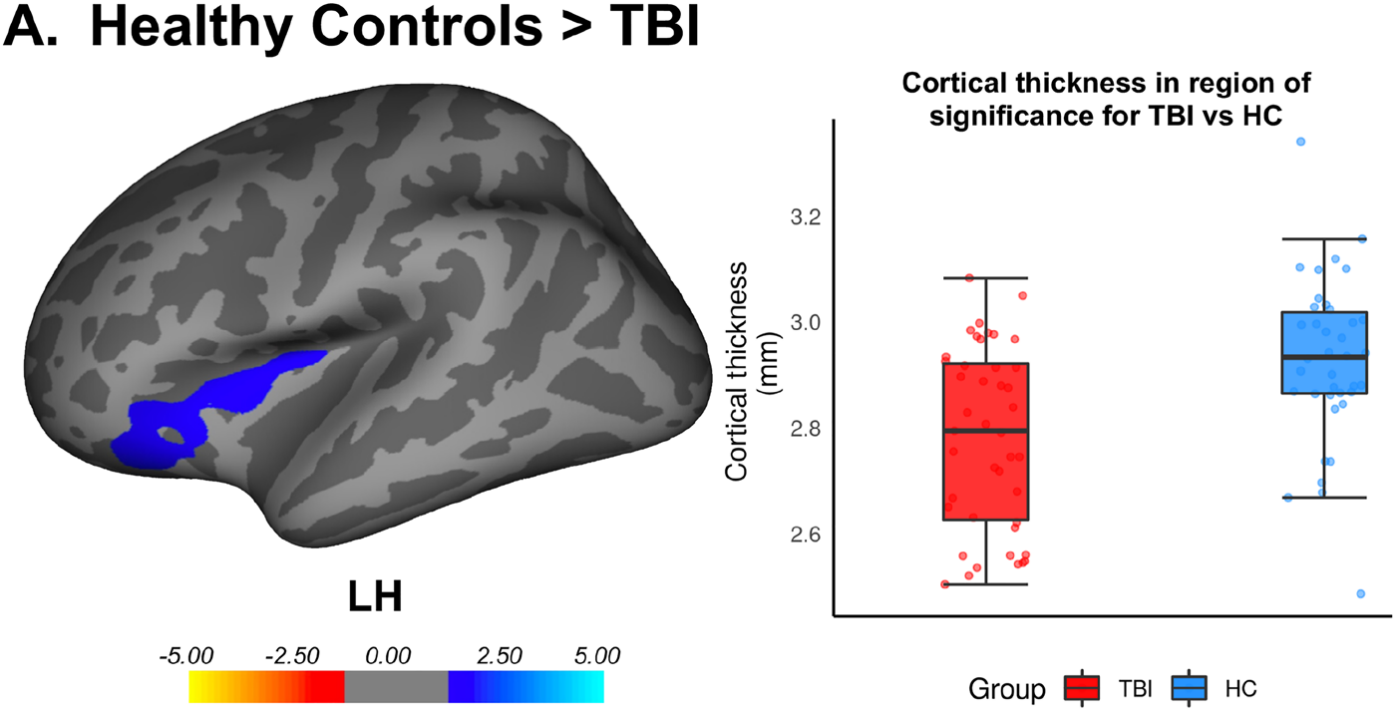
Lower cortical thickness was evident for individuals with TBI compared to healthy controls. (**A**) The regions in which the TBI group had lower thickness were in the left lateral orbitofrontal cortex, pars triangularis, and insula. Panel right—boxplots overlaid with individual datapoints extracted from the Freesurfer output comparing the average cortical thickness in significant cluster between the TBI group (red) and healthy controls (blue). *Note*: Cortical surfaces have been inflated. The color bar was generated following the Monte–Carlo simulation to correct for multiple comparisons. Clusters were threshold at ± 1.3 to indicate *p* < .05.

Differences in cortical thickness were evident between the TBI group and healthy controls. In comparison to healthy controls, the TBI group displayed reduced cortical thickness in the left hemisphere spanning the lateral orbitofrontal cortex, pars triangularis, and insula (Table 1; Figure 2). In contrast, there were no areas where healthy controls had lower cortical thickness than the TBI group.

**TABLE 1:** Significant clusters detected in the left and hemisphere

	Hemisphere	Cluster No.	Max	Vtx Max	Size (mm^2^)	MNI *X*	MNI *Y*	MNI *Z*	CWP	CWP Low	CWP High	Annotation
Healthy controls > TBI—cortical thickness	L	1	3.565	141,498	1330.35	−34.1	−7.3	13.2	0.01770	0.01600	0.01940	Insula
TBI—cortical thickness × face accuracy	L	1	−2.415	60,229	1756.69	−18.4	21.1	57.0	0.00479	0.00360	0.00599	Superior frontal
	R	1	−2.979	44,352	1480.49	7.5	12.1	62.3	0.01792	0.01554	0.02030	Superior frontal
Healthy controls—cortical thickness × face accuracy	L	1	3.988	47,859	6079.22	−13.9	−91.5	22	0.00020	0.00000	0.00040	Superior parietal
		2	3.272	41,681	2321.91	−41.2	−0.7	−37.2	0.00020	0.00000	0.00040	Inferior temporal
		3	2.056	125,001	1182.94	−52	13.3	12.2	0.03784	0.03450	0.04136	Pars opercularis
	R	1	3.284	87,292	3137.32	21.8	−98.1	9.7	0.00020	0.00000	0.00040	Lateral occipital
Healthy Controls—cortical thickness × scene accuracy	L	1	3.747	87,598	1954.73	−7.3	−89.7	29.1	0.00080	0.00040	0.00140	Superior parietal
		2	2.265	21,618	1396.33	−29.7	−87.7	−2.8	0.01236	0.01037	0.01435	Lateral occipital
Healthy control > TBI—cortical thickness × face accuracy interaction	L	1	2.809	74,396	2777.57	−10.8	−75.9	19.7	0.00020	0.00000	0.00040	Cuneus
		2	2.419	50,118	1549.91	−18.5	−63.7	61.3	0.01355	0.01157	0.01157	Superior parietal
		3	3.533	10,965	1404.30	−42.1	−2.4	−37.3	0.02445	0.02168	0.02721	Inferior temporal
	R	1	4.617	62,766	2655.55	16.7	−99.6	−7.5	0.00020	0.00000	0.00040	Lateral occipital
		2	2.726	128,781	2059.41	319	−14.1	59.3	0.00140	0.00080	0.00200	Precentral
		3	3.424	59,863	1791.35	9.4	−54.0	61.9	0.00479	0.00360	0.00599	Superior parietal

*Note*: L = left hemisphere; R = right hemisphere; Max = maximum –log10(*p* value) in the cluster; VtxMax = vertex number at the maximum; size (mm^2^) = surface area (mm^2^) of cluster; MNI X = MNI 305 coordinate of the maximum for *x* direction; MNI Y = MNI 305 coordinate of the maximum for *y* direction; MNI Z = MNI 305 coordinate of the maximum for *z* direction; CWP = *p* value of the cluster; CWPLow = lower 90% confidence interval for CWP; CWPHi = higher 90% confidence interval for CWP; Annotation = annotation of segmented region as defined by Freesurfer.

### 3.3 TBI group and healthy controls show different structure‐function relationships

**FIGURE 3:**
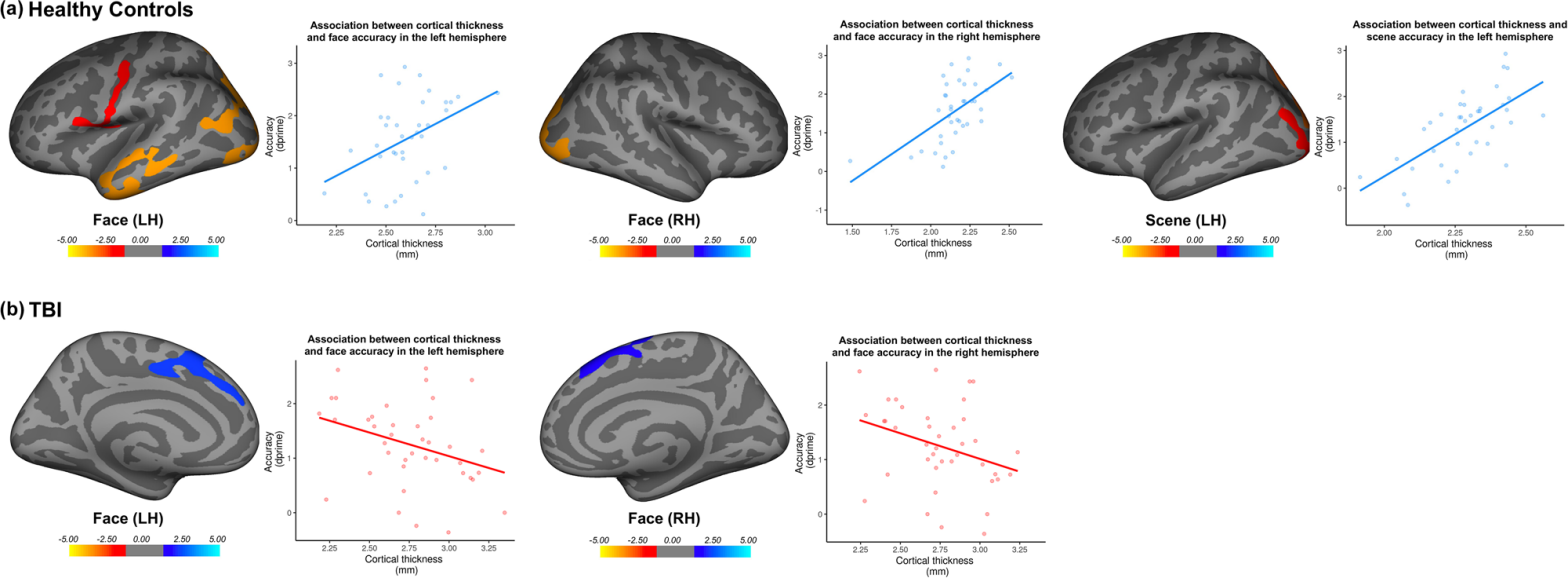
Regions displaying an association between cortical thickness and accuracy in the episodic memory task for healthy controls and the TBI group. Yellow and blue colors indicate positive and negative correlations, respectively. (**A**) Poorer accuracy for faces was associated with lower cortical thickness in left frontal, parietal, and temporal areas, and right occipital areas for healthy controls; poorer accuracy for scenes was associated with lower cortical thickness in left occipital areas for healthy controls. (**B**) Poorer accuracy for faces was associated with greater cortical thickness in superior frontal areas in both the left and right hemisphere for the TBI group. Panel right of brain figures—scatterplots show individual datapoints of the average cortical thickness in the significant clusters as extracted from the Freesurfer output. Positive associations between cortical thickness and memory performance were apparent for healthy controls (blue) while the TBI group demonstrated opposite (i.e., negative) association (red).

Before ascertaining whether differences in cortical thickness and memory performance differed between the TBI group and healthy controls, we first examined structure‐behavior relationships within groups. In healthy controls, poorer accuracy was associated with lower cortical thickness in bilateral occipital regions as well as left parietal and temporal areas for faces, and in left parietal and occipital areas for scenes (Table 1; Figure 3a). Healthy controls did not show an association between cortical thickness and accuracy for animals.

In the TBI group, an association between cortical thickness and memory performance was only apparent for faces (Table 1; Figure 3b). In contrast to healthy controls, however, poorer accuracy was associated with *greater* cortical thickness in bilateral superior frontal regions for the TBI group. The TBI group did not demonstrate any association between cortical thickness and accuracy for scenes or animals.

### 3.4 The association between cortical thickness and memory performance depends on stimulus type

**FIGURE 4:**
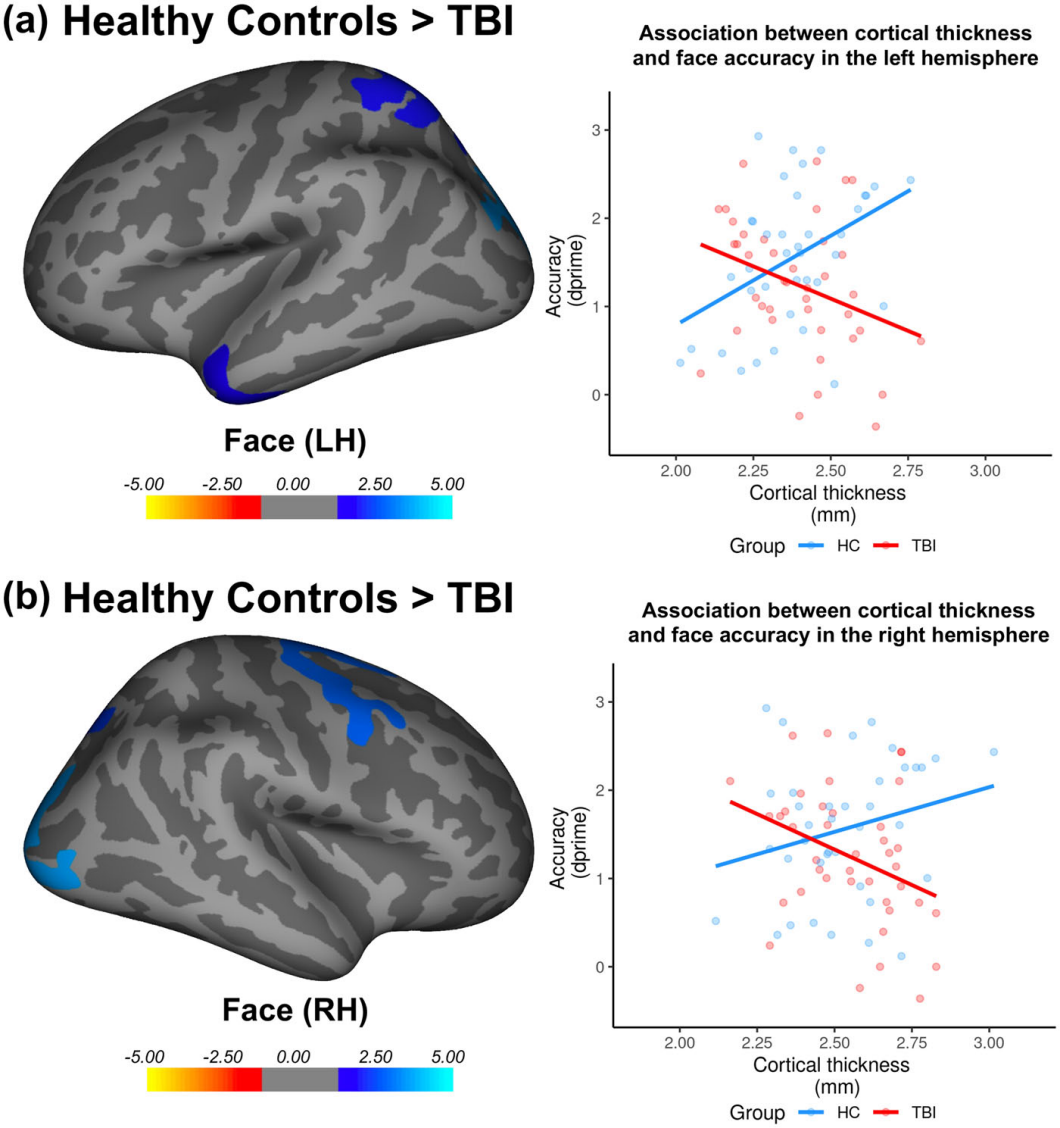
Regions displaying an association between cortical thickness and accuracy for faces in the episodic memory task between groups. (**A, B**) Poorer accuracy for faces was associated with lower cortical thickness in left parietal, temporal, and occipital areas and right frontal, parietal, and occipital areas for healthy controls in comparison to the TBI group. Panel right—scatterplots with individual datapoints of the average cortical thickness in the significant clusters as extracted from the Freesurfer output. While a positive association between cortical thickness and accuracy for faces was apparent for healthy controls (blue), the TBI group demonstrated opposite (i.e., negative) associations (red).

Next, we conducted group comparisons to investigate whether the relationships between cortical thickness and episodic memory performance differed between the TBI group and healthy controls. Interestingly, only the association between cortical thickness and accuracy for faces differed between groups (Table 1; Figure 4). For healthy controls, poorer accuracy was associated with lower cortical thickness in left parietal (i.e., inferior and superior parietal cortices and precuneus), temporal (i.e., inferior, middle, and superior temporal gyri and temporal pole), and occipital areas (i.e., cuneus, lateral occipital cortex, lingual gyrus, and pericalcarine cortex), and right frontal (i.e., middle and superior frontal gyri, paracentral gyrus, and precentral gyrus), parietal (i.e., superior parietal), and occipital areas (i.e., cuneus, lateral occipital cortex, lingual gyrus, and pericalcarine cortex). In contrast, the TBI group showed an inverse relationship whereby poorer accuracy for faces was associated with greater cortical thickness in these regions.

## 4 DISCUSSION

In the current study, we tested the following predictions in individuals with moderate‐severe TBI: (1) episodic memory deficits will differ depending on stimulus type, and (2) the association between cortical thickness and memory will vary depending on stimulus type. Our behavioral results support the notion of category‐specific impairments: the TBI group had impaired accuracy for faces and scenes, but not animals. Moreover, a key finding was that only the association between cortical thickness and accuracy for faces was significant between groups. Poorer accuracy for faces was associated with lower cortical thickness in frontal, parietal, temporal, and occipital areas for healthy controls; however, an unexpected finding was that an inverse (i.e., negative) effect was evident for the TBI group.

Consistent with previous studies that have reported structural changes following TBI (Bigler et al., 2002; Govindarajan et al., 2016; Santhanam et al., 2019; Wang et al., 2015), we found that the TBI group had relatively lower cortical thickness in left frontal regions encompassing the lateral orbitofrontal cortex, pars triangularis, and insula. This finding is not unexpected given that the frontal lobes are located in close proximity to the bony protuberances of the skull and therefore are highly vulnerable to acceleration‐deceleration forces present during injury (Barlow, 2013; Daneshvar & McKee, 2015). Furthermore, cortical thickness was associated with behavioral performance on the episodic memory task. Although such findings have been previously reported in several studies (McCauley et al., 2010; Merkley et al., 2008; Palacios et al., 2013), here we show for the first time in individuals with TBI that this pattern depended on the type of episodic stimuli.

Only the association between cortical thickness and accuracy for faces was significant for both groups. This finding suggests that memory for faces may be more vulnerable to changes in cortical thickness compared to scenes and animals, given that this effect was observed for both healthy individuals and the TBI group. The larger network of cortical regions involved in processing faces could increase their vulnerability to neurological disruption (Haxby et al., 2002; Hoffman & Haxby, 2000; Kesler et al., 2000). Furthermore, it is worthwhile to note that the regions demonstrating an association between cortical thickness and accuracy for faces did not overlap for the TBI group and healthy controls. Whereas these regions were apparent in face‐relevant areas for healthy controls (e.g., temporal and occipital regions), only superior frontal areas were evident for individuals with TBI. In comparison to healthy controls, it may be that memory performance for the TBI group is predominantly driven by memory subprocesses (e.g., encoding) that are subserved by the frontal lobes (Eichenbaum, 2017), and not processes that relate to face processing per se (e.g., perceptual processing).

Critically, group comparisons revealed that only the association between cortical thickness and accuracy for faces differed between the TBI group and healthy controls in several regions (i.e. frontal, parietal, temporal, and occipital areas). This is not unexpected given the involvement of widespread cortical areas in various aspects of face processing (Haxby et al., 2002; Hoffman & Haxby, 2000; Kesler et al., 2000). For example, previous work in patients as well as healthy individuals have shown that learning and recognition for faces is not only supported by “core” occipitotemporal structures such as fusiform and occipital face areas (Mundy et al., 2013; Taylor et al., 2007), but also parietal regions (Leube et al., 2003). Therefore, one explanation as to why only the association for faces differed between groups is that processing of these stimuli is mediated by a network of structures. Given that network dysfunction has been increasingly recognized as a hallmark feature of TBI (Sharp et al., 2014), it is not surprising that faces, but not scenes or animals, most strongly differentiated the TBI group and healthy controls.

A key finding was that the relationship between cortical thickness and memory performance differed between healthy controls and the TBI group. The expected positive association between cortical thickness and accuracy for faces was apparent for healthy controls; however, an inverse (i.e., negative) association was evident for the TBI group. That is, individuals with TBI who had greater cortical thickness were those who had poorer face recognition memory—suggesting that injury‐related processes may have shifted the expected structure‐behavior relationship seen in healthy individuals. The mechanisms driving this finding is unclear; however, one hypothesis is that the TBI group may have encoded faces differently from healthy controls. Indeed, in a recent study from our group (Taing et al., 2021), these same TBI participants demonstrated aberrant brain activity during encoding of faces. An alternative explanation for this finding is synaptic pruning—the process in which inefficient synapses and neurons are removed (Kanai & Rees, 2011). Indeed, lower cortical thickness has been associated with superior IQ (Shaw et al., 2008) and executive functioning (Weise et al., 2019) in healthy young adults. Although synaptic pruning predominantly occurs during adolescence (Tamnes et al., 2010; Toga et al., 2006), it has been established that this process can also be triggered via microgliosis, or increased microglial cells reactivity, following TBI (Ramlackhansingh et al., 2011). Therefore, it is possible that lower cortical thickness was associated with better face recognition memory for the TBI group since inefficient synapses and neurons that may be damaged after injury were removed (Kanai & Rees, 2011).

One previous study has examined the relationship between cortical thickness and episodic memory in individuals with TBI (Palacios et al., 2013). That study reported a positive relationship between cortical thickness in the superior frontal and parietal cortices and memory performance—a finding that differs from those found here. However, a key methodological difference between the two studies was the learned material: verbal versus visual. One explanation is that the changes in structure‐behavior relationship following injury depend on the stimulus type. Therefore, an interesting direction for future research would be to investigate whether the modality in which the information is presented as well as other stimulus categories elicit different structure‐behavior associations.

From a theoretical perspective, the current study provides novel evidence to suggest that there are category‐specific memory impairments following TBI—a finding not supported by some memory models (Squire et al., 2007). Rather, our results align with the emergent memory account which posits that specific medial temporal structures have distinct mnemonic processes (Graham et al., 2010). According to this model, different anatomical regions are thought to subserve perceptual and memory processes for specific categories of stimuli. Importantly, our behavioral and structural results are highly consistent with this account, with findings from both modalities indicating that the most robust category‐specific impairment was for faces following TBI. Understanding that episodic memory is differentially impaired in this population also has important implications for clinical practice. For example, based on our findings, complex visual stimuli such as faces may be more sensitive to deficits in episodic memory, given the higher specificity of such stimuli in distinguishing impaired performances between individuals with TBI and healthy controls.

Some limitations of our study should be considered. One important limitation was that structural images were acquired from different scanners. The use of multisite scanners can lead to differences in image intensity (i.e., contrast of the images) due to difference in magnetic strength as well as other measurement‐related factors (e.g., pulse sequence or hardware components). Although measurement variability in cortical thickness across different scanners with the same field strength has been reported as low (Han et al., 2006), we mitigated such effects by including scanner site as covariate in our analysis. Another limitation is that although semi‐automated approaches, such as those used here, have been established as highly reliable and precise in estimating cortical metrics in healthy individuals (Iscan et al., 2015; Liem et al., 2015), the presence of gross structural injuries can cause inaccuracies in cortical thickness estimations (Irimia et al., 2014). In our study, individuals with focal lesions were also included and therefore we ensured that visual inspections were carefully carried out and any errors (e.g., pial surface errors) were manually corrected.

In summary, our behavioral and structural results provided preliminary evidence indicating memory impairments following TBI are differentially affected depending on the stimulus category. These findings highlight that cortical thickness modulates episodic memory for specific categories of stimuli. Taken together, our study findings have important theoretical and clinical implications with respect to our understanding of episodic memory and processes that may contribute to episodic memory impairments in individuals with TBI.

## CONFLICT OF INTEREST STATEMENT

The Authors declare that there is no conflict of interest.

### PEER REVIEW

The peer review history for this article is available at https://publons.com/publon/10.1002/brb3.3012.

## Supporting information

Supplementary Table 1 Demographic and clinical information of participants
Supplementary Table 2 Clinical characteristics of TBI participants
Supplementary Figure 1 Lesion overlay map of TBI participants on T1 template in MNI space.
Supplementary Figure 2 Regions displaying association between cortical thickness and face accuracy (overall) in the episodic memory task between groups.

## Data Availability

All data supporting the findings of this study can be requested from the corresponding author.
