# Supplementary material for: Traumatic brain injury alters the relationship between brain structure and episodic memory
Source: Brain Behav. 2023 May 3;13(6):e3012. doi: 10.1002/brb3.3012 (PMC10275516; doi:10.1002/brb3.3012)
Supplement: Supplementary file 1 — Supplementary Table 1 Demographic and clinical information of participants Supplementary Table 2 Clinical characteristics of TBI participants Supplementary Figure 1 Lesion overlay map of TBI participants on T1 template in MNI space. Supplementary Figure 2 Regions displaying association between cortical thickness and face accuracy (overall) in the episodic memory task between groups. [file BRB3-13-e3012-s001.docx]

**Traumatic Brain Injury Alters the Relationship Between Brain Structure and Episodic Memory**

Abbie S. Taing^1,2^, Matthew E. Mundy^1^, Jennie L. Ponsford^1,2^, Gershon Spitz^1,2*^

^1^ Turner Institute for Brain and Mental Health, School of Psychological Sciences, Monash University, Clayton, Victoria, Australia

^2^ Monash Epworth Rehabilitation Research Centre, 185-187 Hoddle Street, Richmond, Victoria, Australia.

# Supplementary

**Supplementary Table 1 Demographic and clinical information of participants**

| **Demographic variables** | **Traumatic brain injury,**  **Mean (SD)** | **Healthy controls,**  **Mean (SD)** |
| --- | --- | --- |
| Age (years) | 40.77 (16.46) | 40.05 (17.14) |
| Sex (male/female) | 31/12 | 26/12 |
| Education (years) | 14.23 (2.94) | 14.68 (2.79) |
| Time since injury (months) | 11.07 (11.57) | - |
| PTA (days) | 26.88 (28.06) | - |
| GCS (lowest) | 9.19 (4.23) | - |

GSC = Glasgow Coma Scale; PTA = post-traumatic amnesia. *Note*: Acute GCS were available for *n* = 42 TBI participants; PTA duration were available for *n* = 41 TBI participants.

**Supplementary Table 2 Clinical characteristics of TBI participants**

| **Age (years)** | **Sex** | **Cause**  **of injury** | **PTA (days)** | **GCS (lowest)** | **Time since injury (months)** | **CT/MRI finding** |
| --- | --- | --- | --- | --- | --- | --- |
| 41 | F | Car accident | 19 | 10 | 0.8 | NAD |
| 38 | F | Motorcycle | 7 | 3 | 1 | SAH, SDH |
| 62 | M | Fall of mower | 7 | 14 | 1.2 | Contusion, EDH, SAH |
| 32 | M | Car accident | 22 | 12 | 6 | Contusion, ICH, SAH |
| 48 | M | Motorcycle | 5 | 14 | 3.9 | NAD |
| 45 | M | Bicycle vs. Car | 21 | 8 | 0.7 | SAH |
| 33 | F | Fall of bicycle | 22 | 6 | 1 | SAH |
| 52 | M | Car accident | 14 | 8 | 1.4 | Contusion |
| 21 | F | Pedestrian vs. Car | 3 | 13 | 1.4 | EDH, ICH, SAH |
| 19 | F | Car accident | 63 | 3 | 6.6 | Contusion, EDH, ICH, SAH |
| 22 | M | Car accident | 43 | 15 | 1.8 | SDH |
| 19 | M | Car accident | 24 | 3 | 1.1 | DAI, ICH |
| 19 | M | Car accident | 50 | 3 | 2.7 | DAI, ICH |
| 73 | F | Car accident | 22 | 4 | 2.6 | EDH, SAH, SDH |
| 56 | M | Motorcycle | 23 | 10 | 2.1 | ICH, SAH, SAH |
| 65 | M | Car accident | 24 | 10 | 1.2 | DAI, ICH |
| 32 | F | Car accident | 18 | 11 | 2.3 | ICH |
| 27 | F | Car accident | 7 | 14 | 3.1 | SAH |
| 24 | M | Motorcycle | 39 | 3 | 1.6 | DAI, IVH, SAH |
| 42 | M | Bicycle vs. Truck | 8 | 14 | 1.1 | ICH, SAH, SDH |
| 18 | M | Car accident | 28 | 6 | 1.4 | Contusion, SAH, SDH |
| 18 | M | Pedestrian vs. Bus | 22 | 7 | 1.8 | ICH |
| 62 | M | Motorcycle | Unknown | 15 | 3.4 | SHD |
| 50 | M | Motorcycle | 18 | 13 | 1.2 | ICH |
| 41 | M | Pedestrian vs. Tram | 30 | 6 | 2.5 | ICH |
| 67 | M | Bicycle vs. car | 9 | 8 | 26.93 | DAI, ICH |
| 25 | F | Fall from horse | 34 | 6 | 34.82 | ICH |
| 67 | M | Car accident | 7 | 10 | 19.33 | ICH |
| 42 | M | Car accident | 180 | 4 | 28.34 | DAI, SAH |
| 46 | M | Motorcycle | 15 | 13 | 23.31 | DAI |
| 46 | M | Pedestrian vs. car | 33 | 15 | 13.35 | NAD |
| 50 | M | Motorcycle | 43 | 6 | 26.17 | Contusion, DAI, ICH |
| 62 | M | Car accident | Unknown | Unknown | 28.57 | Pneumocephalus |
| 44 | M | Motorcycle | 34 | 8 | 15.75 | ICH, pneumocephalus, SAH |
| 61 | M | Motorcycle | 23 | 14 | 15.42 | NAD |
| 23 | M | Car accident | 14 | 13 | 17.19 | Contusion, ICH, SAH |
| 20 | F | Pedestrian vs. car | 41 | 3 | 27.58 | Contusion, SAH, SDH |
| 64 | M | Motorcycle | 46 | 9 | 33.70 | DAI, ICH |
| 28 | F | Car accident | 11 | Unknown | 17.42 | ICH |
| 27 | F | Bicycle vs. tram | 21 | 13 | 21.17 | Contusion, SDH |
| 31 | M | Motorcycle | 24 | 14 | 34.82 | ICH |
| 49 | M | Pedestrian vs. car | 10 | 8 | 16.73 | ICH, SDH |
| 42 | M | Car accident | 18 | 3 | 21.34 | ICH, SAH, SDH |

GSC = Glasgow Coma Scale; PTA = post-traumatic amnesia; NAD = no abnormality detected; SAH = subarachnoid haemorrhage, SHD = subdural haemorrhage, EDH = extradural haematoma; ICH = intracerebral haemorrhage, DAI = diffuse axonal injury. *Note*: PTA duration were available for *n* = 41patients; acute GCS were available for *n* = 42 patients.

**Detailed MRI Preprocessing**

The T1-weighted (T1w) image was corrected for intensity non-uniformity (INU) with N4BiasFieldCorrection (Tustison et al., 2010), distributed with ANTs 2.2.0 (Avants, Epstein, Grossman, & Gee, 2008), and used as T1w-reference throughout the workflow. The T1w-reference was then skull-stripped with a Nipype implementation of the antsBrainExtraction.sh workflow (from ANTs), using OASIS30ANTs as target template. Brain tissue segmentation of cerebrospinal fluid (CSF), white-matter (WM) and grey-matter (GM) was performed on the brain-extracted T1w using FAST (FSL 5.0.9; Zhang et al., 2001). Brain surfaces were reconstructed using recon-all recon-all (FreeSurfer 6.0.1; Dale et al., 1999), and the brain mask estimated previously was refined with a custom variation of the method to reconcile ANTs-derived and FreeSurfer-derived segmentations of the cortical grey-matter of Mindboggle (Klein et al., 2017). Volume-based spatial normalization to two standard spaces (MNI152NLin6Asym, MNI152NLin2009cAsym) was performed through nonlinear registration with antsRegistration (ANTs 2.2.0), using brain-extracted versions of both T1w reference and the T1w template. The following templates were selected for spatial normalization: FSL\u2019s MNI ICBM 152 non-linear 6th Generation Asymmetric Average Brain Stereotaxic Registration Model (Evans, Janke, Collins, & Baillet, 2012), ICBM 152 Nonlinear Asymmetrical template version 2009c (Fonov, Evans, McKinstry, Almli, & Collins, 2009).

Many internal operations of fMRIPrep use Nilearn 0.6.1 (Abraham et al., 2014), mostly within the functional processing workflow. For more details of the pipeline, see <https://fmriprep.org/en/latest/workflows.html>.

**
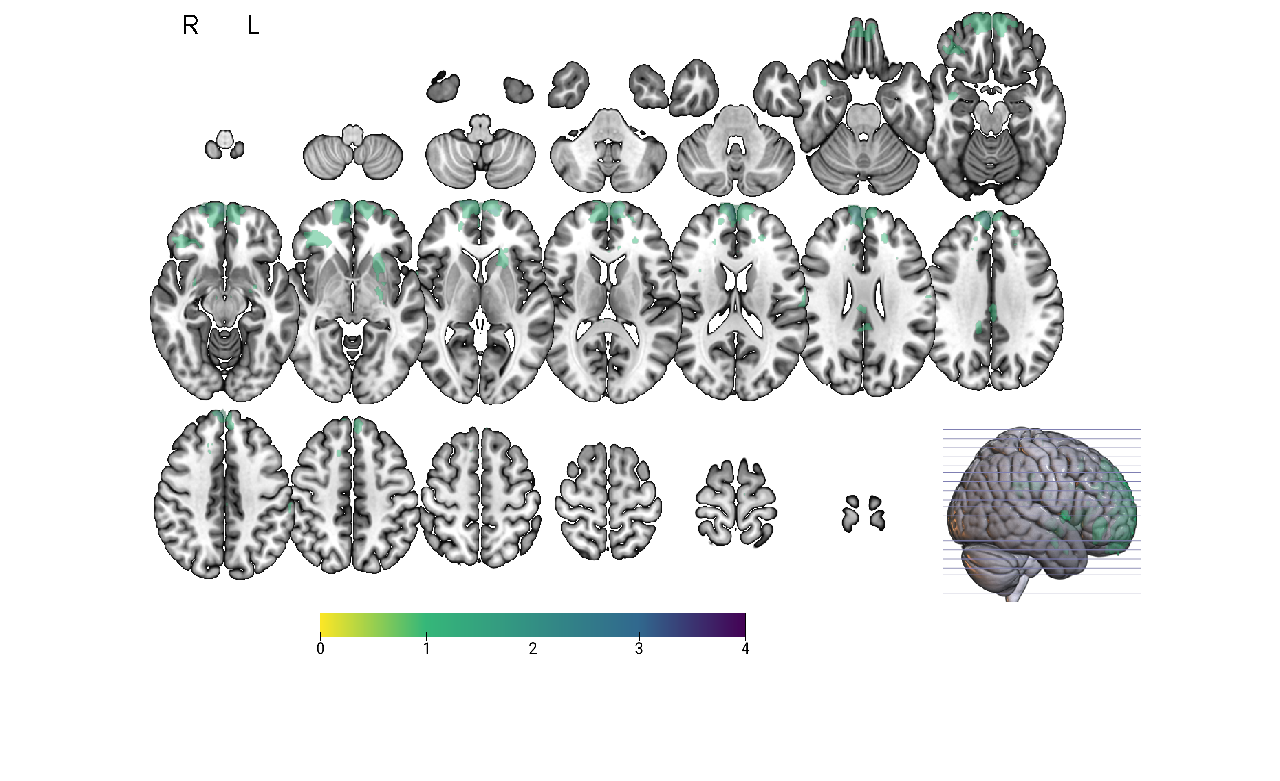
**

**Supplementary Figure 1** **Lesion overlay map of TBI participants on T1 template in MNI space.** *Note*: purple colour indicates greater lesion overlap across participants.

**Differences in sex does not predict memory performance**

Given the differences in the number of male and female participants in the study, we conducted an additional analysis (adding sex as fixed effect into the linear mixed model) to determine whether sex affects memory performance. Overall, we found that sex did not significantly predict memory performance (*t*(223), 95% CI [-0.49664 – 0.03717, *P* = 0.092).

**Episodic memory (overall) and cortical thickness**

The behavioural results reported in the main text were for the first presentation run only. Additional analyses using overall performance (i.e. first and second presentation runs combined) revealed similar findings to those reported in the main text. Overall, the TBI group demonstrated significantly poorer accuracy than healthy controls (95% CI [-0.52 – -0.01]; *P =* 0.030). However, post-hoc analyses revealed significant difference in accuracy only for faces (95% CI [-0.53 – 0.02]; *P =* 0.033), but not scenes (95% CI [-0.49 – 0.06]; *P =* 0.059) or animals (95% CI [-0.33 – 0.22]; *P =* 0.338).

Similar to the main text, group comparisons examining the relationship between cortical thickness and memory performance revealed that a significant association was only evident for faces between groups. Whereas poorer accuracy on face was associated with lower cortical thickness in the bilateral parietal and occipital areas for healthy controls, the opposite pattern was apparent for the TBI group (i.e. poorer accuracy was associated with greater cortical thickness).


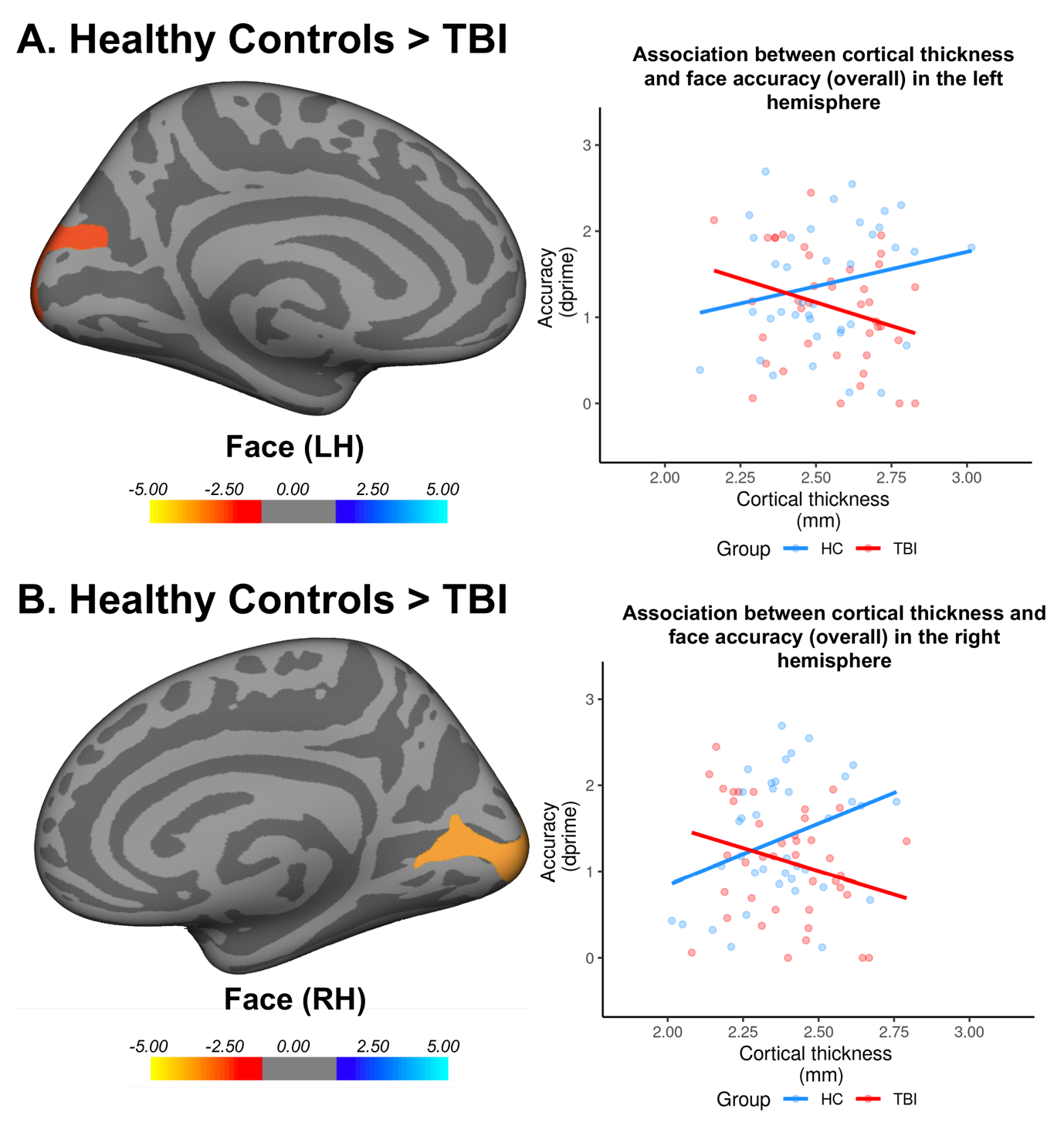


**Supplementary Figure 2 Regions displaying association between cortical thickness and face accuracy (overall) in the episodic memory task between groups. (A – B)** Poorer accuracy for faces was associated with lower cortical thickness in bilateral parietal and occipital regions for healthy controls in comparison to the TBI group. Panel right – scatterplots with individual datapoints of the average cortical thickness in the significant clusters as extracted using the Freesurfer output. While a positive association between cortical thickness and face accuracy (overall) were apparent for healthy controls (blue), the TBI group demonstrated opposite (i.e. negative) associations (red).
